# Pennsylvania State Dental Society

**Published:** 1889-11

**Authors:** J. C. White

**Affiliations:** Sewickley, Pa.


### Reports of Society Meetings.

### PENNSYLVANIA STATE DENTAL SOCIETY.—(*Continued.*)

### THE YOUNG PRACTITIONER.

BY J. C. WHITE, D.D.S., SEWICKLEY, PA.

It was with the greatest reluctance that I finally consented to occupy a small portion of your valuable time in the reading of a paper. Much more should I have enjoyed myself, had I been privileged to sit in this convention, a guest and an appreciative listener; not because I want to shirk the work, for a man who is not willing to give some of his time and energy to uplifting the standard of our profession does not deserve the honor that is conferred upon him when he is named as a member of a society.

It would be presumptuous for me to discuss a subject with the idea that it would give enlightenment to my fellow-practitioners. My experience has been far too limited for that; and yet, limited as the time has been, I have been enabled to observe a number of things of which I was entirely ignorant until I began to practise. From the time a young man opens his office the critical eye of the public is upon him, ever ready to pick out his imperfect points, which, as we all know, at this time are very numerous; and, on the other hand, they are very reluctant to laud him for what he is too apt to think was a most remarkably skilful operation. It is true, the operation may not have been a difficult one, and if an older man had performed the same, he might not have thought it anything more than every-day practice; but with the young man it is different, and though all others fail to see what he considers a good point, he does not, and will be so pleased over his success that he will be frequently found talking about it, much to the disgust of those with whom he comes in contact, to say nothing of the annoyance caused the one upon whom the operation was performed. Above all things, the operator should refrain from talking about his patients. Not infrequently we find the narrow-minded rival looking upon and treating his young colleague as though he were an intruder and it was very audacious for him to think of locating near him without first consulting His Royal Highness. Do not think, gentlemen, for a moment that I consider this the general rule. By no means. I say occasionally you find this, and I am glad to say it is principally confined to small towns. In the city and among the more educated class they are found to be much more congenial. Education and broad-mindedness go hand in hand. There is no room for doubt that the young practitioner frequently thinks his education is far more extensive, and that he is infinitely more skilful than his fellow-practitioner. In fact, he is so far advanced when he receives his diploma that the different meetings of the local societies and the State dental societies are looked upon by him as intended solely for the older but less educated men of his profession. Then comes the "quack," with his large gilt sign hung half-way across the sidewalk,—"Teeth extracted and filled without pain; plates made and inserted while you wait; best plates only five dollars." All of this is, of course, disgusting to the better class of our profession, and a great amount of work comes to us after the patient has been in the hands of these men. Their work is not only injurious to the patient, but it conveys a wrong impression upon the public in general, and we of course suffer from it. The work we build up they tear down; the good we may do they undo, and so the seesaw is kept up.

But why is it we have so many "quacks?" When do they become so, and how is their number to be lessened? Is it when they have been in practice a number of years, and have succeeded in building a good practice among the better class of citizens, or is it in the early days of their practice, when they are ambitious, not so much to do good work among the better class as to get a great number of patients regardless of the class? This seems to be the height of their ambition. No; I hold it is a rare exception for one to depart from the legitimate practice of a dentist after he has been in practice a number of years; it is when he first starts out on his professional career that he is at all inclined to advertise extensively, and thereby get a large practice.

Then, the "quack," while he may have little or no effect upon the practice of the older members of the profession, has a decided effect upon the young dentists. It should be the aim of every man to do honest work, never allowing himself to become so hurried that he will slight in any particular the work he has undertaken. This I take to be the principle upon which each young man starts out, to spend the time necessary to do this work faithfully. He must charge a sufficient amount to justify him for the work he has done. His views and those of his patients will frequently differ widely on this point, the young man holding tenaciously to the point that if his work is done as good as it is possible to do it, he must be paid as much as his more experienced colleague.

The patient, on the other hand, uses the silly argument that he must wait until his hair is white with age before he thinks of charging such a figure. "Why," says the patient, "I can go across the way, to Dr. ——" (doctor, by the way, is so frequently misapplied in these cases that it almost makes me long to be called mister again), "and he will do the work for one-half or one-third what you ask." Then the young operator must explain the why and wherefore of this; and, although his arguments may be very good, it is not always sufficient to convince the average patient until after he or she has been through the hands of the neighboring quack. We are glad to say this, however, is almost invariably sufficient to convince one that he would have been better off if he had followed the advice given. While I strongly advocate the theory of a young man charging a good figure, I caution him against excessive charges. In this he should be as honest as he is in his work. Now, gentlemen, since the quack has come, and since it is the young man who begins this practice, is it not our duty, would it not be to our advantage, to keep a closer eye upon the younger men of our profession?

The time has come when no undergraduate is permitted to practise in our state; and yet, sad it is to relate, the graduate himself is often found lowering the dignity of his profession to that of a trade. I do not overlook the fact that many who start out with little or nothing feel that they are compelled to advertise in order to get a start in the world; then, when they have once started in this way, and find that the peers of the profession in their locality pay no attention whatever to them, do not advise them against their course, and they are not invited to join the local societies, they lose all hope of ever becoming identified with these men, and finally are satisfied to remain where they have started. No, gentlemen; while the young man who becomes a quack is largely to blame, the responsibility rests somewhat on the leading members of the profession.

After graduation each young man should be induced to join some good local society. If this is run properly and he receives sufficient encouragement, there is little danger of his ever becoming a quack. Do not think I speak from personal experience; I have every reason to be grateful for the encouragement and good-will I

have received at the hands of my fellow-practitioners. I feel a life-long indebtedness to the members of the Pittsburg Dental Association for the kindly manner with which I was received into their midst, but I feel others were not so fortunate, and it is for these that I intercede.

Not only must we educate our patients to the fact of the importance of our work and of the vast improvement that has been made in our profession, but we must teach our own young men that we are no longer the "tooth-pullers" of a few years ago, but that we now pose as the tooth-savers of the nineteenth century. We are no longer classed among the tradesmen of our country, but have risen to the rank of a scientific profession. Its future success depends not upon the man whose hair has grown white in the service of his profession; whose eye is dimmed with the approach of old age; his work is almost done. It has been the labor of such men as these that has placed the standard where it now rests, but its future success remains with the young.

### DISCUSSION ON DR. J. C. WHITE'S PAPER ON "THE YOUNG PRACTITIONER."

*Dr. J. C. Green.*—A few of us can remember the time when the "tooth-pullers" in the small villages looked at each other as they passed along the street, and did not want to see one another. They would not recognize each other. I have some reason to remember something of this kind; and when I commenced the practice of dentistry, a few years ago, I made it a point to see that every young man who came into our town was taken by the hand and welcomed. I pursued that course, and to-day every man that has come into our place is a particular friend of mine. They are all nice men, and in our little town of several thousand inhabitants we now have seven or eight dentists, and every man is a graduate. Occasionally a man drops in, not wanting to see you. They fear the advancement. The old idea was, "I do not know you, and don't want to see you." Now the idea is to take hold of every man and encourage the young men, and I fully endorse what the essayist has said, that the older men must encourage the younger.

*Dr. L. A. Faught.*—I can fully endorse the paper that has been read, but wish to add a few words. There is a relationship which the young practitioner holds other than as a practitioner of dentistry in the following of his calling. I think it is not unfrequently the case, even at the present day, that when one patient goes from one operator to another, and sees an older member into whose hands he has passed, and says this work was done by so and so, what do you think of it? he does not express an opinion favorable to it. When told the dentist's name he says, "I do not know him." When he meets that gentleman down-town, however, he says, "How do you do, doctor?" He does know him. It is a relationship that ought not to exist.

There is another relationship, the one the young practitioner holds to dental meetings. We cannot expect the work of older men to continue on indefinitely unless we have new blood. We cannot expect to engraft that just when we need it. We must bring them in, encourage them, and make them feel they are of importance upon the dental floor. If we could do that, when the older members who have been the life of the society drop the mantle, it will then be taken up by those who have become imbued with the work. As an illustration of this, seven out of the nine papers here are prepared and read by young men,—men whose names, with one or two exceptions, have not appeared in the society. They come here, and we have encouraged them, and I am sure that at this stage of the meeting we can all attest that the papers have been well presented, and there has been that friction that brings out the truth. We have an evidence of the good of that kind of work.

*Dr. H. E. Roberts.*—Regarding helping the young practitioner along, a great deal depends on the young man himself. It was brought to my mind very forcibly a short time ago. One of my college class-mates was a lady. She came to me a week ago, and said, "I am not treated right in any way." I said, "Well doctor, have you gone around and tried to mingle with dentists?" She said, "No." I said, "You do that, and see if the dentists do not give you a helping hand." A little while afterwards I found her name on a committee on some dental work, and it was all right with the dentists. I think as much depends upon the young men as the help from the older ones. It should be mutual. No one is more willing to help the young practitioner than I am.

### DISCUSSION ON DR. A. P. BRUBAKER'S PAPER ON "REFLEX EFFECTS OF DENTAL IRRITATION."

*Dr. L. A. Faught.*—I am delighted that we have a paper of this class to bring before the association. We cannot too highly impress upon the minds of all, old and young, the necessity of being familiar with the anatomical, physiological, and pathological relations of the nervous system and the teeth. There is much yet to be learned in this respect. I am thoroughly satisfied, from what little knowledge I have of physiological conditions, what a great benefit it has been to me in the treatment of cases that have come under my care. The more extended our knowledge the better we can serve our patients. What I wish to say is entirely commendatory of the paper brought before us.

*Dr. E. C. Kirk.*—It is not necessary for me to add anything by way of endorsement of the paper; I fully concur with Dr. Faught. I want, however, to call attention to a few cases in this line which I think will bear out the views of Dr. Brubaker. I had a boy under my care for the treatment of his teeth. About six months afterwards, when all question of dental irritation from that source was eliminated, he was suddenly taken one day at table with an attack of lock-jaw,—spasmodic. He was brought to me twenty-four hours afterwards by his mother, and his family were particularly alarmed, for he was having these attacks repeatedly every two or three weeks. In connection with his family physician, I tried to discover some cause of irritation. I found that the second bicuspid on the lower left side had not erupted, and could not possibly get through, because of lack of space between the sixth-year molar and the first bicuspid; as I recollect, the sixth-year molar was devitalized,—at least it was defective,—and my advice was to extract it, and thus give room for the bicuspid to erupt. Under ordinary circumstances I would not have extracted it. The sixth-year molar was extracted, and the second bicuspid in the course of time came through and filled up the space reasonably well, and his attacks of lock-jaw ceased almost immediately after the extraction of the molar. Whether it was the sixth-year molar or the mal-erupted bicuspid I don't know; at any rate a cure followed extraction.

In the earlier part of my practice one of my first cases,—and one which made an impression upon me—was that of a girl, who came into my office suffering with pain in the sixth-year molar. It was absolutely without defect, except a slight cavity in a sulcus. The pain was definitely located in this tooth by her. She also had pain in the ear,—acute otalgia,—and she was frantic in her demands that the tooth should be removed. I told her it was not the source of her trouble. I did not know as much about the subject then as now, but I thought it was neuralgia from some source situated near that tooth. She was evidently suffering terribly, and I said, "As long as you are sure it is this tooth, I will destroy the pulp and not extract it." This done, however, the pain continued. I then did what should have been done at the commencement,—went over each tooth and examined every surface. I had gone completely around the upper jaw, also the lower, until I came to a wisdom-tooth partially through. I passed a probe over the crown, examined the distal aspect, and discovered an exposed pulp. The tooth was extracted and the pain ceased.

At another time I had brought to me a lady who was suffering with a chronic middle-ear catarrh. As I was talking with her husband in regard to reflex dental disturbances, he said, "I thought the matter had some dental origin." The upper and lower wisdom-teeth on that side were in position, and perfectly sound. My experience, however, in a number of cases led me to look upon these teeth as a source of much ear-difficulty, particularly catarrhal, and, as they were of no value to her, I advised extraction, thinking possibly there was some growth upon the roots,—exostosis. I extracted the two teeth, and found them both extensively exostosed, and the cure of her catarrhal difficulty followed immediately upon their removal,—hearing being restored almost immediately after extraction.

I reported quite recently in the *Cosmos* a case of an impacted cuspid tooth, where coincident with this neuralgic difficulty was a defect in the ocular muscles, leading to divergent squint. I could trace no relationship between the tooth and the eye difficulty, and considered them as merely coincident, but the eye trouble was removed by extraction of the tooth.

I have been led to believe that the acute attacks of otitis children have at the period of eruption of the permanent teeth are largely due to dental irritation from the breaking down of the deciduous set, or exposure of the pulp, or from abscess, etc., and my experience bears out the statement that Dr. Brubaker has made in his paper, that where the reflex disturbance is not great it appears to be a peripheral irritation. Where the irritation is slight, as in exostosed roots, the expression in the ear is chronic and fixed, and of a low degree of inflammation. I merely mention these cases as following out the thought of Dr. Brubaker.

*Dr. C. S. Beck.*—I am very glad to hear such a paper, and thankful that the gentleman is with us; and I would like to give the history of one or two interesting cases where there was some doubt as to the source of irritation. One was in a lady who had been wearing an upper denture for a number of years, and who was affected with twitching of the left eye. She consulted several physicians, and they went over the muscles and nerves of the face, and examined the uterus, to see if there was any trouble there. After a close investigation they could not decide what caused this constant twitching. The eye was sometimes quite red and inflamed. She was in my office one day, and, in mentioning the subject, she said, "I am sure all my teeth are out, having worn a plate for five years. Still, I have an unpleasant feeling in my mouth sometimes, and cannot account for it." The gums looked a little red at one point, caused by the rubber plate. I inserted a delicate probe, and struck a hard substance like enamel. I said, "Are you sure you have had every tooth removed?" "Yes," she said, "I have them at home." I said, "There is something there, and this may be the exciting cause;" and suggested that she have her family physician come and administer an anæsthetic, and let me remove it. She was quite plucky, though, and said, "You can do it now." I made a crucial incision, and found a cuspid tooth lying horizontally across the jaw, which I removed with considerable difficulty. It seemed a cross between a supernumerary and normal tooth, and the little root had an exostosis upon it as large as a good-sized buckshot. I saw her the next day, and she said she was perfectly relieved. The inflammation had gone down, and the eye ceased to twitch. This had been going on for considerable time.

Now an amusing case. I had a young man in one Saturday evening who was suffering with a terrible pain in the first molar in the upper jaw. I examined it, and found it perfectly sound, but observed that there was a wisdom-tooth in the lower jaw in which the nerve was exposed. I tried to convince him that the sixth-year molar was not the seat of pain. He said, "I want that tooth out," referring to the first molar. I extracted it, and he was satisfied. Sunday morning he came up to the office and asked, "Is the man in who pulls teeth?" I extracted, at his demand, the second molar, and here were two teeth sacrificed. A little while later he returned and exclaimed, "My God, it has gone down here!" pointing to the wisdom-tooth. I removed this, and the pain ceased.

The final case I will mention is that of a lady who was *enceinte*, and whose physician feared there would be a premature birth. Her husband said she had suffered more or less from her teeth. She was brought to my office, and in going over her teeth on the lower left side I found a wisdom-tooth embedded, and the moment I touched it she started. Ether was administered, the tooth removed, and all the uterine pain ceased.

*Dr. Dickie.*—A case comes to my mind that occurred in London. A man of position and wealth, who had consulted the best medical skill possible, was afflicted with acute rheumatism of the hipjoint, which had resisted all efforts for seven years. At one time attention was called to a fang in the inferior maxilla, which was troubling him, but not sufficiently marked to excite suspicion. That he might not suffer with the root in connection with the hip-joint trouble, a dentist was summoned, the fang removed, and the instant it left the jaw the hip-joint trouble ceased and never returned.

*Dr. J. C. M. Hamilton.*—A lady in our vicinity became violently insane for a year, the malady taking the form of religious mania of a very violent type. She was wearing an artificial lower denture, but her upper teeth were natural. She thought one day she must have a tooth pulled, and would have no peace until this was accomplished. She came into my office, and I saw it looked perfectly sound. I took hold of it, and, finding it slightly loose, I concluded it would be a good thing for the woman to remove it, and I undertook to extract it. I do not think I ever saw such a tooth to pull, and finally got an assistant to hold her head. It was exostosed so much that it was the size of a large hickory-nut, and the moment I took out the tooth she became rational, and has been so ever since.

Another case was that of a prominent gentleman in our place. In operating upon his teeth, several years ago, the pulp-canals were found to be ossified. He finally took a trip out to California, and while there was afflicted with a pain in the back of his head. He went twice to Philadelphia and once to New York for consultation, and returned home without having received any benefit, and then decided to consult a dentist. I told him the condition in which I found the pulps of the two teeth, and said the only remedy I knew of was to remove the teeth. This was done, some eight teeth being extracted, and he never had any pain afterwards. The pulp-canal of each tooth was ossified, and all in the same jaw.

*Dr. W. E. Magill.*—Another phase of the subject will be best illustrated by just saying a word or two regarding a case in our vicinity. The patient, a farmer, is afflicted with a periodic neuralgic pain, always in the facial region. It is an annual pain that comes on during the harvest season, and he is entirely relieved during the autumn and winter. He has exhausted the skill of the region around, and each new physician to whom he has gone has advised him to have some tooth extracted. One of the forms in which this attack comes on is in this way. He may be out driving when he is suddenly taken with a spasm, the reins falling from his hand. Whenever the attack comes on he decides to have a tooth extracted, and he has for the time being temporary relief, which is generally accredited to the prescription of the new physician; but when the next season comes on he has the same trouble for the same period. The point I wish to make is this: if we are not competent to embrace the whole field in our diagnosis, we may do the very thing the physician does,—extract the tooth with no benefit to the man. He has lost several teeth in this way, is now nearly edentulous, and this summer has the same old trouble again. I see the point that the paper makes,—the necessity for a larger extension of our study, and for a better comprehension of the sympathies which exist between one part of the system and another.

*Dr. J. C. Green.*—I think we have gotten a better idea of the extensive influence of reflex dental irritation, and we are indebted to the doctor for his paper. In this connection I would simply say that when I was a boy, a few years ago, in my neighborhood there was an old lady, a relative of mine, whose jaws became locked. She went for treatment to the city of Wilmington, and the physician advised her to go to Philadelphia. She went to an eminent practitioner there, and he said, "Go to a dentist and have a tooth removed, and the jaws will open." Medical men, after all, have sometimes shown us a few things, and demonstrated that they know a few things, and we are very anxious to have medical men give us their experience, and are willing to respect their opinions once in a while.

*Dr. W. H. Fundenberg.*—Sometimes they know something and sometimes they do not. I had come to me two cases which physicians had treated for weeks and finally sent the patient to the dentist, and in both these cases, upon questioning closely, it was developed that the toothache was a neuralgic pain. They had not gone far enough.

I remember a case that occurred with my father. A patient came to him to have filled a left superior cuspid tooth, and on account of the small space for operating he decided to drive a wedge between, to separate the teeth. Immediately upon this the lady felt a sharp pain in the eye, resulting in an almost total blindness, which lasted for several days. It gradually grew better, her sight was restored, and the tooth was afterwards filled.

*Dr. C. S. Beck.*—One more case. A lady suffered two years with a disease that baffled her physicians. They said to the husband they could not detect the cause of the trouble, and that there was no hope of saving the lady's life; she might live for years, and perhaps not. The case was abandoned, and the lady made as comfortable as circumstances would admit. She thought she would like to have her teeth made more comfortable, and to this end they took her to a celebrated dentist, and he said, "Madam, your mouth is in a terrible condition; you are suffering very much from poison. The condition of the gums and tissues of the mouth indicate mercurial poisoning. Your health is poor, and I think if these fillings and some of these teeth were removed, your health would be much improved." This excited the husband very much, and consultation was had with the attending physician. The result was the removal of a certain number of bad amalgam fillings and also a certain number of teeth, and the lady's health was regained.

DISCUSSION ON PAPER BY DR. JAS. S. KING, OF PITTSBURG, ON "TREATMENT OF EXPOSED TOOTH-PULP."

At the conclusion of his paper, Dr. King said, "I would like to say that, in looking over the proceedings of last year, I find Dr. Kratzer said, according to the record, that I combined with creosote a small portion of oil of cloves. That I have never done."

*Dr. C. V. Kratzer.*—It was a mistake on my part, then.

*Dr. G. W. Klump.*—I am much obliged to the gentleman for his paper. I would like to ask him in what way he tells when the pulp is living or on the point of dissolution; in other words, how he prepares his cavity before he uses the capping, and does he entirely expose the pulp or leave a thin layer of dentine over it?

*Dr. Jas. S. King.*—As a rule, I leave in the bottom of the cavity some of the disintegrated dentine; but there are many cases that come under my treatment where actual exposure and perhaps continued pain have been present for a week, month, or possibly six months. I do not know whether I exactly understand your question as to the preparation of the cavity. I remove all the disintegrated tooth-structure that is possible. If strangulation has taken place there is no restorative treatment that we can offer. In some cases I may perhaps make an incorrect diagnosis, and put in a capping when it would be better not to make it.

*Dr. G. W. Klump.*—It seems an open question to me as to what is disintegrated dentine. I notice in your remarks that you say you remove all softened disintegrated dentine. There is a fine question in point. I think it is important in saving pulps to remove all the softened dentine; in other words, to find out the condition of the pulp. I think when pulps have been painful and have assumed a pathological condition, after a time they recede in the pulp-chamber, and when you cap a pulp in that condition, with any part of the covering over it, you have a vacuum there, and if anything abhors a vacuum it is a pulp. It is better to expose it entirely and fill up the space. That is my experience, and I would like to hear from some others.

*Dr. Jas. S. King.*—Did I understand you to say that a pulp not entirely exposed becomes enlarged, swollen, and congested?

*Dr. G. W. Klump.*—No, there is a recession.

*Dr. Jas. S. King.*—Can it recede without exposure? I have been practising the method I described for a little over eighteen years, and I cannot recollect any cases where I have had trouble from recession of the pulp. I hesitate in no instance to cover the pulp if I imagine the course will justify it, whether the exposure be large or small, and rarely have I failures. It may seem a boast to say it, but it is nevertheless true. If there is a recession of the pulp, you will of course have a vacuum, but I have never had any experience that taught me that vacuums have given trouble.

*Dr. Jesse C. Green.*—I would like to ask Dr. King whether, after the application of the preparation to the pulp, it is followed by pain,—say one, two, or six months,—is the pulp alive after that?

*Dr. Jas. S. King.*—Followed by pain? Yes. In cases where the exposures are large there is a measure of uncertainty. If I make a mistake, and cover a pulp where actual strangulation has intervened, the life is cut off and there can be no restoration.

*Dr. Jesse C. Green.*—I meant to ask whether the pulp generally lived when treated as described.

*Dr. Jas. S. King.*—If the exposure has existed for a considerable time,—painful for months and years,—and even of large area, and I am sure that vitality is unimpaired to any great extent, I feel that I have no cause not to adopt my usual treatment. It is only in cases of doubtful vitality, and where there is a possibility of my judgment being in error, that I hesitate.

*Dr. C. V. Kratzer.*—Will Dr. King tell us what kind of paper he uses?

*Dr. Jas. S. King.*—It is a thin and very stiff paper, manufactured in Pittsburg. Any paper that is free from dirt is good. The method of treating this paper in chloride of zinc is known only to the manufacturers. In my first experience I used a thin septum of vulcanite. This had some disadvantages, but was nevertheless successful. This chloride-of-zinc paper has been very successful in my hands, and I know of nothing to equal it.

*Dr. C. V. Kratzer.*—As to my statement published in the report of the last meeting, Dr. King was reported at that time by a member as using a preparation composed of equal parts of carbolic acid and oil of cloves, mixed with oxide of zinc, and I thought to correct it by giving what I understood to be his formula,—equal parts of oil of cloves and pure wood creosote, combined with oxide of zinc, with which I must say I have been reasonably successful. I have had some failures, but few. I have perhaps used finer discrimination than Dr. King has, for I have frequently given the pulp the benefit of the doubt and the devitalizing paste.

*Dr. C. S. Beck.*—I may not be right, but I think Dr. Jack was the originator of the treatment with creosote and oil of cloves in combination. While I was very much pleased with Dr. King's paper, I must take some issue with the gentleman. I do not think that the capping of pulps in all cases is such a success. I do not dispute Dr. King's success. I think we often overlook doubtful cases, because we think as the tooth is comfortable the pulp is intact. I must also differ with my friend, Dr. Klump. One point Dr. King made, and that was that if the decay was very shallow, and the pulp exposed, it indicates to us a large pulp-chamber, and that he takes this preparation and places paper over the pulp-exposure, and then he introduces his hard filling. Now I cannot see how that hard filling can be introduced over an oily substance like creosote and oil of cloves, and be made a success; and even if a success, I should think it rather disastrous treatment, due to the fact that thermal changes would in time destroy the pulp.

The main success I have had in pulp-treatment has been in placing the pulp in as comfortable a position as possible, introducing a temporary material,—gutta-percha,—and letting that filling remain for a year or eighteen months; and after that, if I feel satisfied that I can fill that tooth with a hard material, I do so, but even then have some little doubt as to the success of the work. A pulp often takes a long time to die. I think a pulp uncovered and exposed to the secretions of the mouth will die rapidly. After an inflamed condition has been set up I do not see how it can be reduced,—how that engorged condition can be brought down. In my own experience I doubt whether there is as large a degree of success as is claimed. While they claim it in honesty, there are a great many failures they know nothing of.

Another point: Dr. Klump says he removes every portion of decay over an exposed pulp. I do not. I think it is good to remove all you can, leaving, if possible, a very thin stratum of devitalized dentine,—acting upon the theory advanced by many in our profession, that the lime-salts have been removed only from the semi-decalcified dentine, and that in time these salts will be reestablished. I think very often the best we can do is to leave this thin stratum, providing we can thoroughly disinfect it. You know that this devitalized matter is largely inhabited by microorganisms, and to get rid of these is the first step towards success. I really think that the old theory, however, when a pulp has been exposed any length of time,—to devitalize it, thoroughly disinfect the canal, and introduce in place of the pulp a good root-filling,—is about the best practice after all.

*Dr. Wm. B. Miller.*—I wish to coincide with Dr. Beck, for my experience is like his. While I wish to keep the pulp alive as long as possible, a great majority of us make a mistake in trying to keep alive those that ought to be destroyed. There is a difference between actual and partial exposures. Whenever a pulp has a thin layer of dentine covering it, it is not an exposure, and does not indicate a capping, but the placing of a material that will prevent thermal changes. Oftentimes we have patients come to us saying they have been suffering agony for some time, and that Dr. So-and-so capped the pulp. I have frequent experiences of that kind. Many pulps should be devitalized that are indiscriminately capped.

*Dr. G. W. Klump.*—My only object in entirely uncovering the pulp is to find out whether there is any secretion there. When a pulp has not given any trouble I do not pretend to expose it. I speak only of those pulps that have ached. If there is a large exposure, I think it would be folly to cap it without filling up the vacuum. You may find it dead, but they very rarely die without giving some trouble. I never expose a pulp unnecessarily; but when it has given trouble I am very careful to find out the condition it is in.

*Dr. J. A. Mayer.*—In my experience I would not hesitate to cap a pulp that had never ached or given trouble; but if it has been painful, and the exposure of long duration, I would not think of treating it in that manner until I first understood the conditions, and was sure the treatment offered a reasonable hope of success.

*Dr. W. B. Miller.*—I disagree with Dr. Beck in the use of gutta-percha over the pulp.

*Dr. C. S. Beck.*—This is confined simply to pulps that have given no trouble. In excavating, if I find that the pulp is exposed, I remove as much of the dentine as I think I dare, and thoroughly disinfect the cavity. Two years ago I was very much censured by a gentleman because I said I used bichloride of mercury, one grain to the ounce, as a disinfectant. I have yet to see the slightest bad results from such treatment. After preparing my cavity thoroughly, I leave as much of the dentine as I think I ought to; I then try to destroy the micro-organisms by disinfectants. After having done that I use any proper covering. I use a small cap of platinum filled with the following paste made of one part wood creosote, one part oil of cloves, a little oxide of zinc. The latter acts as a vehicle to hold my creosote and cloves. This treatment, I believe, Dr. Jack introduced, and I have used it for years. I mix my paste thin, so it will flow nicely, fill that little cap full, and place it over the orifice. I wait ten, fifteen, or twenty minutes, until that becomes thoroughly set, and after that I remove all the superfluous margin, so that there is not a particle of this cement projecting from the cavity. In that cavity I place my gutta-percha filling.

*Dr. Jas. S. King.*—I thought in my paper I kept up the distinction between treating exposed pulps and those covered by a thin layer of dentine, as mentioned by Dr. Miller. Where there is a thin septum of dentine we are not treating an exposed pulp. It may be painful; but when it has been painful from acids, etc., it is not an exposure. When I spoke of exposed pulps, I meant it in the literal sense. I cannot conceive how Dr. Miller could remove the disintegrated dentine, and finally remove the last film, and not know that without pain he had a dead pulp.

*Dr. W. B. Miller.*—There are many cases, when we come to remove the entire covering from the pulp, that we find oozing out a small drop of pus, the suppuration only occurring at the point of exposure, and the lower two-thirds still alive. The fact of pus having formed is the reason why we have not pain. That portion is dead.

Subject passed.

(To be continued.)

